# The ‘Reducing Psychosis Risk by Targeting Trauma’ Trial: Protocol of a Feasibility Randomised Controlled Trial of Trauma‐Focused Cognitive Behavioural Therapy and Eye Movement Desensitisation and Reprocessing Therapy for People With At‐Risk Mental States

**DOI:** 10.1111/eip.70095

**Published:** 2025-10-04

**Authors:** Filippo Varese, Kim Cartwright, Amanda Larkin, Marina Sandys, Aidan Flinn, Alice Newton, Jasmine Lamonby, Mica Samji, Clare Holden, Samantha Bowe, David Keane, Nadine Keen, Amy Hardy, Debra Malkin, Richard Emsley, Kate Allsopp

**Affiliations:** ^1^ Division of Psychology and Mental Health School of Health Sciences, University of Manchester, Manchester Academic Health Science Centre Manchester UK; ^2^ Complex Trauma and Resilience Research Unit Greater Manchester Mental Health NHS Foundation Trust Manchester UK; ^3^ Manchester University NHS Foundation Trust Manchester UK; ^4^ Psychosis Research Unit Greater Manchester Mental Health NHS Foundation Trust Manchester UK; ^5^ South London and Maudsley NHS Foundation Trust London UK; ^6^ Department of Psychology Institute of Psychiatry, Psychology & Neuroscience, King's College London London UK; ^7^ Lancashire and South Cumbria NHS Foundation Trust Preston UK; ^8^ Department of Medical Statistics and Trials Methodology Institution Institute of Psychiatry, Psychology and Neuroscience, King's College London London UK

**Keywords:** ARMS, CBT, CHR, EMDR, psychosis, RCT, trauma, UHR

## Abstract

**Background:**

Trauma exposure is pervasive in people with an At Risk Mental State (ARMS) and is associated with adverse clinical and functional outcomes. While promising developments have been made in treating trauma in psychosis, evidence regarding the efficacy of trauma therapies in ARMS individuals is limited. This trial aims to evaluate the feasibility of conducting a future randomised controlled trial (RCT) to determine the efficacy of Eye Movement Desensitisation and Reprocessing (EMDR) and trauma focused cognitive behavioural therapy (TF‐CBT) in people with ARMS.

**Method:**

Seventy ARMS individuals with a history of trauma will be randomised to receive 24 sessions of EMDR plus treatment as usual (TAU), 24 sessions of TF‐CBT+TAU, or TAU alone. Feasibility will be determined against pre‐specified thresholds for recruitment, retention, treatment engagement, and fidelity. To examine the promise of efficacy of EMDR and TF‐CBT, participants will complete a battery of clinical and mechanistic measures at baseline and 9‐month post‐randomisation, including assessments of attenuated psychotic symptoms and post‐traumatic symptoms. Clinical notes will be reviewed to identify transitions to first episode psychosis up to 12 months post‐randomisation. Qualitative interviews with trial participants, therapists, and professional stakeholders will explore the acceptability of EMDR and TF‐CBT and factors to facilitate future implementation of trauma therapies in routine practice.

**Conclusions:**

If a large‐scale RCT is deemed feasible, it will be possible to establish whether EMDR and/or TF‐CBT represent beneficial treatments to augment existing evidence‐based care for individuals at ultra‐high risk for future psychosis, potentially reducing transition rates and improving clinical outcomes for ARMS individuals.

## 1 Introduction

Past research has confirmed that traumatic life events can increase the risk of developing psychosis. Meta‐analytic findings suggest that exposure to various potentially traumatic events in childhood (e.g., bullying, sexual and physical abuse, etc.) is associated with a threefold increase in psychosis risk, with a population attributable risk of 33% (Varese et al. 2012). In parallel, a growing number of mediational and mechanistic studies have identified a range of psychological impacts of trauma that are plausibly implicated in the apparent association between trauma and various symptoms of psychosis (e.g., Williams et al. 2018; Alameda et al. 2021). This suggests that the emotional and psychological consequences of trauma may represent tractable treatment targets to reduce psychosis liability in at‐risk individuals with a history of trauma.

Recent systematic review and meta‐analyses have confirmed that trauma exposure is pervasive in individuals with At‐Risk Mental States (ARMS; Yung et al. 2005) and/or similar criteria for identifying individuals at ultra‐high risk for psychosis (e.g., Kraan et al. 2015). Trauma exposure in ARMS individuals is linked to comorbidities associated with heightened risk for psychosis transition, including poorer social and occupational functioning and more severe attenuated psychotic symptoms (Mayo et al. 2017; Brew et al. 2018; Peh et al. 2019). However, current treatments for preventing psychosis do not sufficiently target the impacts of trauma, and recent reviews have highlighted this as a critical area for future research to enhance treatment outcomes in this at‐risk group (Zarubin et al. 2023).

Several evidence‐based therapies specifically target the consequences of trauma, particularly trauma‐focused cognitive behavioural therapy (TF‐CBT) and eye movement desensitisation and reprocessing (EMDR). These therapies have a strong evidence base for treating post‐traumatic stress symptoms (NICE 2018) and are increasingly applied to other mental health sequelae of trauma, including psychosis (Hardy et al. 2024). Recent meta‐analyses have confirmed that these trauma therapies can improve post‐traumatic stress and, to a lesser extent, psychotic symptoms in people with psychosis (Brand et al. 2018). Yet, these promising developments have not led to parallel innovation in the treatment of trauma sequelae in people at clinical high risk for future psychosis. To date, early promising findings have been provided by a small RCT of an 8‐session EMDR intervention for people with prodromal psychosis and comorbid post‐traumatic stress disorder conducted in China (*N* = 57; Zhao et al. 2023) and a small non‐randomised trial (*N* = 14; Strelchuk et al. 2024) that offered ARMS individuals an ‘EMDR for psychosis’ intervention previously developed for first episode psychosis clients (Varese et al. 2021; Varese et al. 2024). Further research considering not only EMDR, but also other evidence‐based interventions for trauma (i.e., TF‐CBT) suitably adapted to maximise acceptability and effectiveness in clients with attenuated psychotic symptoms is urgently needed to inform the development of an evidence base for addressing the trauma‐related needs of individuals with ARMS.

## 2 Aims

The primary aim of the current study is to determine the feasibility of conducting a future RCT to examine the clinical efficacy and mechanisms of action of TF‐CBT and EMDR interventions purposely adapted for people with ARMS. Feasibility will be assessed through four key indicators (trial recruitment, trial retention, EMDR/TF‐CBT treatment engagement, and EMDR/TF‐CBT treatment fidelity).

The secondary aims are to (1) examine the promise of efficacy of EMDR/TF‐CBT on important clinical outcomes and the acceptability of assessment instruments to investigate the efficacy and mechanisms of action of EMDR and TF‐CBT in this participant group, (2) gather data on the safety of EMDR and TF‐CBT in people with ARMS, (3) gather qualitative data to explore the acceptability of EMDR and TF‐CBT in trial participants, and (4) gather qualitative data from trial therapists, supervisors, and other professional stakeholders to explore their views on barriers and enablers of successful implementation of EMDR and TF‐CBT in future routine mental health care for the ARMS group.

## 3 Methods

### 3.1 Design

We will conduct a feasibility randomised controlled trial (RCT) with two nested qualitative studies. The trial will be a pre‐registered (ISRCTN76699092) single‐blind parallel group RCT with three arms: EMDR+treatment as usual (TAU), TF‐CBT+TAU, or TAU alone (see CONSORT diagram provided in Appendix S1). Key feasibility outcomes will be assessed by Research Assistants (RAs) at baseline and at a 9‐month post‐randomisation assessment, with further outcomes collected at 12‐month post‐randomisation via screening of medical records.

### 3.2 Participants

This trial will aim to recruit 75 individuals who (1) meet ARMS criteria operationalised using the updated version of the Comprehensive Assessment of At‐Risk Mental States (CAARMS‐23; Yung et al. 2023) and (2) report exposure to past traumatic events in childhood or adulthood on the Trauma and Live Events questionnaire (TALE; Carr et al. 2018). The full inclusion and exclusion criteria of the RCT are listed in Table 1.

**TABLE 1:** Inclusion and exclusion criteria.

Inclusion criteria	16 years of age or older Meeting ARMS criteria as defined by the CAARMS Capacity and willingness to provide informed consent Reported exposure to at least one potentially traumatic life events, as assessed by the TALE
Exclusion criteria	Under 16 years of age Non‐English speaking or requiring an interpreter for the intervention (the assessment battery at present can only be delivered in English) Evidence of recent or past transition from ARMS status to first episode psychosis (FEP), operationally defined as meeting CAARMS transition criteria (i.e., history of a treated or untreated psychotic episode of 1 week's duration or longer) and/or previous or current treatment with antipsychotics at dose of over 5 mg of haloperidol or equivalent for over 3 weeks Judged by the assigned care‐coordinator/responsible clinician and the research team as not sufficiently clinically stable to engage safely in a clinical trial of trauma‐focused therapy (e.g., acutely suicidal and suicide attempt in the previous 2 months; in a current mental health crisis; not in stable housing).

*Note:* ARMS = at risk mental state; CAARMS = comprehensive assessment of at risk mental states (Yung et al. 2005); TALE = Trauma and Life Events checklist (Carr et al. 2018).

Prospective participants will be identified through Early Detection and Intervention Teams (EDIT) and Early Intervention for Psychosis (EIP) services at three UK National Health Service (NHS) mental health trusts in the North West of England, and other mental health services that interface with EDIT/EI services and provide support to individuals who may meet ARMS criteria (e.g., student mental health services; complex primary care psychological therapy services, etc.). ARMS individuals who participated in recent non‐interventional studies by the research team and consented to further contact for research involvement purposes will also be approached. Trained RAs will obtain informed consent prior to an eligibility assessment using the CAARMS‐23 and the TALE.

#### 3.2.1 Randomisation and Allocation Concealment

Participants will be randomly allocated to either (1) EMDR+TAU, (2) TF‐CBT+TAU, or (3) TAU only at a ratio of 1:1:1. Randomisation will be independent and concealed via an online pseudo‐random list with random permuted blocks of varying sizes. Allocation will be concealed from the RAs conducting follow‐up assessments. If an RA becomes “unblinded” to a participant's allocation during the study, where possible, the participant will be allocated to another RA. Where this is not possible, interview‐based measures will be recorded with participant consent, and the measure will be scored by blinded RAs.

## 4 Interventions

### 4.1 Comparator: TAU

TAU will be in line with all standard and individually prescribed clinical interventions as directed by national clinical guidelines for psychosis/ARMS (National Institute for Health and Care Excellence [NICE] 2013, 2014) and the participants' clinical teams. In EDIT services and ARMS pathways within EIP services, support provided to ARMS individuals typically includes active monitoring of prodromal symptoms and the offer of preventive interventions for psychosis, in particular Cognitive Behavioural Therapy. However, TAU for ARMS individuals may differ depending on additional clinical needs and whether service users are referred to and/or managed by other primary or secondary care mental health services over the course of the RCT. In no case will the trial team interfere with teams' clinical decision making by asking to withhold referrals or interventions that would be offered to participants as part of their usual care, including other active psychological interventions. The uptake of interventions comprised in TAU will be documented via reviews of participants' medical notes for up to 12 months post‐randomisation.

### 4.2 EMDR Intervention

Participants allocated to the EMDR+TAU arm will receive up to 24 sessions of EMDR over a 9‐month treatment window, in addition to TAU. Each session will last up to 90 min and will be audio/video‐recorded for fidelity monitoring purposes when participants consent to this. The specific protocol used as part of this trial has already been evaluated and implemented successfully in other feasibility trials conducted by the research team with clients who have already transitioned to FEP (for further details on this treatment approach, see Varese et al. 2021; Varese et al. 2024), which will be gradually refined over the course of this trial to maximize acceptability and clinical benefit for ARMS participants.

### 4.3 TF‐CBT Intervention

Participants allocated to the TF‐CBT+TAU arm will receive up to 24 sessions of TF‐CBT delivered over a 9‐month treatment window, in addition to TAU. Each session will last up to 90 min and will be audio‐recorded for fidelity monitoring purposes when participants consent to this. The TF‐CBT intervention delivered as part of this trial will be adapted for delivery in individuals with an ARMS from a similar, annualised intervention already developed for a definitive trial of trauma‐focused therapy for individuals with schizophrenia‐spectrum disorders by integrating intervention strategies and models for the treatment of PTSD and psychotic symptoms (for further details, see Peters et al. 2022).

### 4.4 Procedures to Monitor and Promote EMDR and TF‐CBT Fidelity and Adherence

The treatment will be delivered by EMDR‐ and CBT‐trained therapists who have experience in working with people with psychosis, ARMS, and complex trauma. All therapists will attend an initial 3‐day training workshop in the EMDR protocol and a separate 3‐day training in the TF‐CBT protocol used as part of the trial. Throughout the study, trial therapists will attend separate EMDR and TF‐CBT supervision sessions with experienced therapy supervisors. Treatment fidelity will be ensured in two ways. First, the EMDR and TF‐CBT supervisors will rate a quasi‐random selection of therapy recordings (for up to 10% of the therapy sessions delivered for each experimental arm of the trial) using the EMDR Fidelity Rating Scale (EFRS; Korn et al. 2017) and the adapted version of the Cognitive Therapy Scale—Revised (CTS‐R) developed by Peters et al. (2022), which will be further modified as needed to capture additional adaptation related to ARMS‐specific work. This will ensure that all therapists involved in the RCT demonstrate at least acceptable adherence to EMDR and TF‐CBT when delivering these therapies to ARMS clients. Secondly, after each therapy session, therapists will be asked to complete a standardised session record form to monitor session content and monitor adherence against the EMDR and TF‐CBT protocols used in the trial.

## 5 Outcomes

The RCT is designed to evaluate the feasibility of conducting a future definitive trial. Hence, the trial's primary outcomes will be the key feasibility indicators required to recommend progression towards a future larger‐scale trial (trial recruitment, trial retention, EMDR/TF‐CBT treatment engagement, and EMDR/TF‐CBT treatment fidelity). These will be operationalised according to pre‐specified feasibility thresholds informed by past research on trauma‐focused interventions in people with first episode psychosis (Varese et al. 2024) and approved by an independent Trial Steering Committee (Table 2).

**TABLE 2:** Principal feasibility outcomes and proposed feasibility thresholds considered in the RESTART feasibility RCT.

Criterion	Critical feasibility outcome	Other feasibility and acceptability data relevant to the criterion		Proposed thresholds on critical outcome
1. Recruitment rate	Number of participants consented into the trial and randomised	Number of referrals per month Source of recruitment Number of participants contacted, number of participants assessed for eligibility Reasons for non‐eligibility or withdrawal of interest	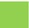 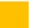 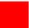	Feasibility will be demonstrated where an average of at least 3 participants are recruited and randomised per month of active recruitment. If at least 2 participants are recruited per month, then a future trial will be feasible but additional strategies must be identified to support recruitment (e.g., informed by other feasibility data relevant to this criterion) If an average of 1 participant is recruited per month over the recruitment period (< 20 participants), feasibility within the current design will not be demonstrated
2. Therapy engagement	% who drop‐out of therapy/% who did not receive treatment allocated	Number of therapy sessions attended Reasons for failed initiation of treatment Reasons for premature termination of treatment Session record forms for each therapy session Analysis of characteristics of therapy engagers vs. non‐engagers Qualitative interviews with SU participants	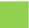 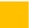 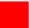	Feasibility will be demonstrated if at least 70% of the participants in the intervention arm completed at least 8 sessions of EMDRp or TF‐CBTp If 50%–70% of participants in the intervention arm complete at least 8 sessions of EMDRp or TF‐CBTp, a future trial considering these intervention will be feasible but changes to the trial design or treatment protocol may be warranted to ensure deliverability (e.g., informed by other feasibility data relevant to this criterion) If less than 50% of participants in the intervention arm complete at least 8 sessions of EMDRp or TF‐CBTp, feasibility within the current design will not be demonstrated
3. Assessment retention	Percentage of participants who are lost to follow‐up	Reasons for withdrawal from the study Qualitative interviews with SU participants	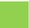 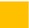 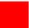	If at least 70% of participants are retained and the 9‐month post‐randomisation assessment, feasibility will be demonstrated If 30%–70% of participants are retained at the 9‐month post‐randomisation assessment, a future trial will be feasible if strategies to overcome barriers are identified (e.g., via other data relevant to this criterion) If less than 30% of participants are retained at the 9‐month post‐randomisation assessment, feasibility within the current design will not be demonstrated
4. Therapy fidelity	Adherence ratings from therapy tapes	Session record form for each therapy session (including reasons for deviation from protocol)	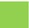 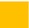 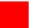	Feasibility will be demonstrated if over 80% of rated therapy tapes will be rated as acceptable If 50%–80% of rated therapy tapes will be rated as acceptable, a future trial will be feasible if strategies to overcome identified barriers (e.g., exploring the reasons for deviation from protocol recorded in the therapist checklists) If less than 50% of rated therapy tapes will be rates as acceptable, feasibility within the current design will not be demonstrated

*Note:* Amber = continue but modify protocol—the future definitive trial is feasible with modifications; Green = continue to main study without modifications—feasible as it is. Red = stop—future definitive trial is not feasible. The feasibility thresholds below have been informed by and adapted from those employed in a past trial of trauma‐focused therapy for psychosis conducted by the Chief Investigator (the EASE trial; Varese et al. 2021; Varese et al. 2024), which were proposed/approved by the EASE trial TSC.

To evaluate the feasibility of completing the battery of measures to be employed in the future definitive trial, and gather outcome data to establish the ‘promise of efficacy’ of EMDR and TF‐CBT, participants will be assessed at baseline and 9‐months post randomisation follow‐up appointments using an assessment battery covering relevant clinical and mechanistic measures (see Table 3 for details). Additionally, at 12‐months post randomisation, participants' clinical notes will be screened to assess transition rates to first episode of psychosis and document the support received as part of TAU over the course of the trial. Additional data collection will be undertaken with all participants allocated to the TF‐CBT + TAU and EMDR+TAU arms of the trial over the course of treatment, and will include (1) sessional measures to monitor symptom fluctuations over the course of treatment (using the PQ‐16 and a sessional measure of post‐traumatic symptoms adapted from Peters et al. 2022); (2) measures of therapeutic alliance (by administering at session 3, session 12 and at therapy completion the Working Alliance Inventory—Short Form Revised; Hatcher and Gillaspy 2006); (3) a brief questionnaire to assess, at the end of treatment, the tolerance/acceptability of the psychological therapy they received (Devilly 2004).

**TABLE 3:** Summary of measures used to assess participant symptoms at baseline and follow‐up.

		Baseline	9‐months
Demographic information and clinical history			
Age, sex, ethnicity, employment status and occupation (if relevant), marital status, education level, disability status, self‐reported diagnosis, treatment history, duration of EDIT/Early Intervention service input if applicable, number and reasons for past psychiatric hospitalisations, current prescribed medications for mental health difficulties (including dosage), diagnosis.		X	Updated as required
At‐risk mental state related measures			
PQ‐16	A short measure designed to assess psychosis risk	X	X
CAARMS‐23	A semi‐structured interview to identify those at ultra‐high risk of developing psychosis	X	X
CAPE‐N	A measure to detect those who present an increased risk for developing psychosis	X	X
Trauma‐related measures			
TALE	A measure designed to assess exposure to adverse and traumatic life experiences commonly reported by people with psychosis/ARMS.	X	—
PCL‐5	A self‐report questionnaire assessing the presence and severity of PTSD symptoms	X	X
ITQ	A brief measure assessing the severity of symptoms of PTSD and complex PTSD as defined in the ICD‐11.	X	X
DSPS	A measure assessing lifetime and past month dissociative symptoms	X	X
TMQ	A questionnaire assessing the quality of the memory of the traumatic event	X	X
PTCI‐9	A brief measure assessing thoughts and beliefs related to/arising from the traumatic event	X	X
MCQ‐PTSD	A questionnaire measuring metacognitive beliefs following a traumatic experience	X	X
PTGI‐SF	A short‐form questionnaire measuring positive growth following adverse life events	X	X
Health economic measures			
EQ‐5D‐5L	A short self‐report measure assessing service users' current health	X	X
EPQ	An adapted version of the EPQ for specific use in services users with ARMS	X	X
Other mental health and functioning measures			
GAD‐7	A commonly used questionnaire assessing symptoms of anxiety.	X	X
PHQ‐9	A commonly used questionnaire assessing symptoms of depression.	X	X
CAS‐1R	A questionnaire measuring cognitive attentional syndrome and metacognitive beliefs	X	X
QRP	A questionnaire measuring aspects of recovery	X	X
AQ‐10	A short measure assessing autism‐spectrum traits	X	—
EoTT	A new questionnaire developed for this trial to assess concerns that participants may have about engaging in trauma focused interventions; an initial validation of the EoTT (e.g., internal consistency and association with other relevant measures collected as part of the trial) will be conducted using data collected as part of this feasibility RCT	X	—

*Note:* Economic Patient Questionnaire (Davies et al. 2007); AQ‐10 = Autistic Spectrum Quotient (Allison et al. 2012); CAARMS = The Comprehensive Assessment of At‐Risk Mental States (Yung et al. 2005); CAPE‐*N* = negative symptoms scale of the Community Assessment of Psychic Experiences scale (Mossaheb et al. 2012); CAS‐1R = Cognitive Attentional Syndrome Questionnaire (Faija et al. 2019); DSPS = The Dissociative Subtype of PTSD Scale (Wolf et al. 2017); EoTT = Expectations of Therapy Questionnaire (under development as part of a PhD at the University of Manchester); EPQ = Economic Patient Questionnaire (Varese et al. 2021); EQ‐5D‐5L = The EuroQol 5‐Dimension 5‐Level measure (Janssen et al. 2013); GAD‐7 = The Generalized Anxiety Disorder Questionnaire (Spitzer et al. 2006); ITQ = International Trauma Questionnaire (Cloitre et al. 2018); MCQ‐PTSD = The Metacognitions Questionnaire for Post‐Traumatic Stress (Wells 2009); PCL‐5 = The PTSD Checklist for DSM‐5 (Blevins et al. 2015); PHQ‐9 = The Patient Health Questionnaire (Kroenke et al. 2001); PQ‐16 = The 16‐items version of the Prodromal Screening Questionnaire (Ising et al. 2012); PTCI‐9 = The brief Post‐Traumatic Cognitions Inventory (Wells et al. 2019); PTGI‐SF = The Post‐Traumatic Growth Inventory short‐form (Cann et al. 2010); QPR = The Questionnaire about the Process of Recovery (Neil et al. 2009); TALE = The Trauma and Life Events checklist (Carr et al. 2018); TMQ = The Trauma Memory Questionnaire (Halligan et al. 2003).

In line with recommended practice (Health Research Authority [HRA] 2023; International Council for Harmonisation [ICH] 2016), all adverse events (AEs) experienced by participants over the course of the RCT will be monitored and assessed by a clinically qualified senior researcher to establish seriousness (i.e., those resulting in death; life‐threatening circumstances; new or prolonged hospitalisations and emergency care; persistent disability or incapacity), severity, relatedness to trial procedures/interventions, and expectedness. All suspected serious SAEs will be independently assessed by the chair of an independent trial steering committee and reported to relevant regulatory bodies. To promote active monitoring of AEs, RAs will inquire about possible serious AEs during all follow‐up assessments with trial participants and via an additional phone call 4.5 months following the end of baseline assessment. Medical notes will also be monitored up to 12 months post‐randomisation to identify any unreported AEs.

## 6 Feasibility RCT Analysis

Descriptive statistics will be used to document participants' flow in the trial in accordance with all relevant fields of the CONSORT statement extension for feasibility trials (Eldridge et al. 2016). These will include: the number of referrals received per month, the source of recruitment, number of participants contacted, number of participants assessed for eligibility, number of participants consented into the trial (and reasons for non‐eligibility or withdrawal of interest, when appropriate and where given), number of participants allocated to each RCT arm (and those who did not receive the treatment allocated as intended), and the number of participants retained at the 9‐month follow‐up assessment. Furthermore, descriptive analyses of EMDR and TF‐CBT engagement data (number of sessions received by participants allocated to the EMDR+TAU and TF‐CBT + TAU arms) and fidelity data (therapy session records forms data as well as EFRS and CTS‐R data) will be conducted to estimate fidelity/adherence of the interventions received by study participants.

To examine the acceptability of assessment measures administered as part of the trial and the viability of extracting transition information and concomitant treatment data from clinical notes, all measures will be examined for completeness and reasons for missingness. There will be no formal hypothesis testing comparing the three groups for clinical effectiveness; however, summary statistics (Mean, SD, % of missing data) will be tabulated for each quantitative measure by trial arm at each time point, and unadjusted mean differences between the arms at the 9‐month follow‐up and their associated 80% CI will be calculated to provide some evidence towards the promise of efficacy of the interventions considered in this feasibility trial and inform the design of the future definitive trial (Lee et al. 2014).

## 7 Nested Qualitative Studies

Following their participation in the RCT, between 18 and 24 participants will be invited to take part in a qualitative semi‐structured interview. Participants will be recruited using purposive sampling to maximise variation in key characteristics (e.g., age, ethnicity, trial arm, levels of engagement with the experimental intervention, therapy completers vs. non‐completers, and drop‐outs). Interviews will be based on a modified version of Sekhon's acceptability framework (Sekhon et al. 2017) to examine the extent to which EMDR and TF‐CBT may be regarded as acceptable options for augmenting the care provided to people with ARMS.

Further qualitative interviews will be conducted with trial therapists, EMDR/TF‐CBT supervisors, as well as mental health professionals from services approached during the feasibility trial to identify prospective participants. The study will gather a qualitative understanding of factors relevant to the acceptability of EMDR and TF‐CBT for ARMS individuals and views on barriers and enablers of successful implementation of these interventions in future routine care for the ARMS group. Interviews will be based on a modified version of Sekhon's acceptability framework (Sekhon et al. 2017) integrating relevant components of Normalisation Process Theory (NPT; May and Finch 2009; May et al. 2018), a widely used theory to explain the processes by which novel interventions become, or fail to become, embedded into routine practice. A total of 12–20 participants will be purposively sampled for maximum variation, with consideration of a range of key characteristics (e.g., professional/educational background, age, and ethnicity, etc.).

Interviews for both studies will be audio recorded, transcribed verbatim, pseudonymised at the point of transcription, and analysed following the National Centre for Social Research Framework approach (Ritchie and Spencer 2002). The findings from this qualitative work will be integrated with the quantitative feasibility outcomes to provide a rich and comprehensive understanding of the trial's viability and to directly inform the final decision regarding progression to a full‐scale RCT.

## 8 Discussion

The evaluation of trauma‐focused psychological therapies in people with psychosis is a recognised research priority (NICE 2014) and an area of growing research and clinical innovation (Hardy et al. 2024), but applications in the context of indicated prevention strategies remain scarce. The overarching aim of this trial is to determine the feasibility of running a definitive trial of EMDR and TF‐CBT to determine the efficacy of these interventions in an ARMS population. Conducting such research is vital to improving outcomes for those affected by prodromal psychotic symptoms and trauma.

Based on extensive evidence linking trauma sequelae to psychotic symptoms (e.g., Williams et al. 2018) and promising findings from interventional studies in China and the UK (Zhao et al. 2023; Strelchuk et al. 2024), it is possible that EMDR and TF‐CBT may lead to significant therapeutic gain on attenuated psychotic symptoms in ARMS individuals who have a history of trauma. Through assessing potential recruitment and retention rates into a future larger‐scale trial, the acceptability of EMDR and TF‐CBT interventions and the extent to which therapists can deliver our adapted trauma‐focused interventions with high levels of fidelity, and qualitative evidence to maximise the implementation potential of these treatments in future routine care, this feasibility RCT will pave the way to more robust evaluations that could augment existing evidence‐based care options for individuals at ultra‐high risk for psychosis.

While this feasibility trial is limited by its sample size, and therefore its inability to establish for definite the effectiveness of EMDR and TF‐CBT in the client group, it features several key strengths compared to previous early‐phase evaluations of trauma‐focused therapy in people at heightened risk for psychosis (Zhao et al. 2023; Strelchuk et al. 2024). These include (1) the use and further refinement of bespoke treatment manuals that have already been successfully delivered in clients with full‐blown psychotic symptoms (Peters et al. 2022; Varese et al. 2024), (2) the concomitant evaluation of multiple active trauma‐focused treatments, increasing the likelihood of identifying potentially beneficial treatments and therefore allowing future practitioners and clients to choose from various treatment options that may better suit individual preferences and circumstances; (3) the recruitment of a diverse range of participants, including both service users within specialist early intervention for psychosis services and other mental health settings that may support individuals with prodromal psychotic symptoms. An additional strength of the study is its assessment of the feasibility of collecting mechanistic measures that, in a future larger‐scale trial, may shed light on the mechanisms of action of trauma‐focused therapies in the ARMS group. This will facilitate the design and delivery of future trials combining both efficacy and mechanistic evaluations of trauma‐focused therapies in this at‐risk population (Dunn et al. 2015). Such trials are needed to further clarify the evidence base on the necessary ‘active ingredients’ of psychological interventions for trauma and (emerging) severe mental health problems.

## Conflicts of Interest

Co‐authors Varese, Bowe, Larkin, Holden, Keane, and Malkin are involved in the delivery of CBT and EMDR training workshops and events. All other co‐authors have no conflicts of interest to declare.

## Supporting information


**Appendix S1:** CONSORT flow diagram.

## Data Availability

Data sharing not applicable to this article as no datasets were generated or analysed during the current study.
