# Supplementary figures and images for: The ‘Reducing Psychosis Risk by Targeting Trauma’ Trial: Protocol of a Feasibility Randomised Controlled Trial of Trauma‐Focused Cognitive Behavioural Therapy and Eye Movement Desensitisation and Reprocessing Therapy for People With At‐Risk Mental States
Source: Early Interv Psychiatry. 2025 Oct 4;19(10):e70095. doi: 10.1111/eip.70095 (PMC12495450; doi:10.1111/eip.70095)

**Supplementary Materials**

**Supporting Information 1**

CONSORT Flow Diagram


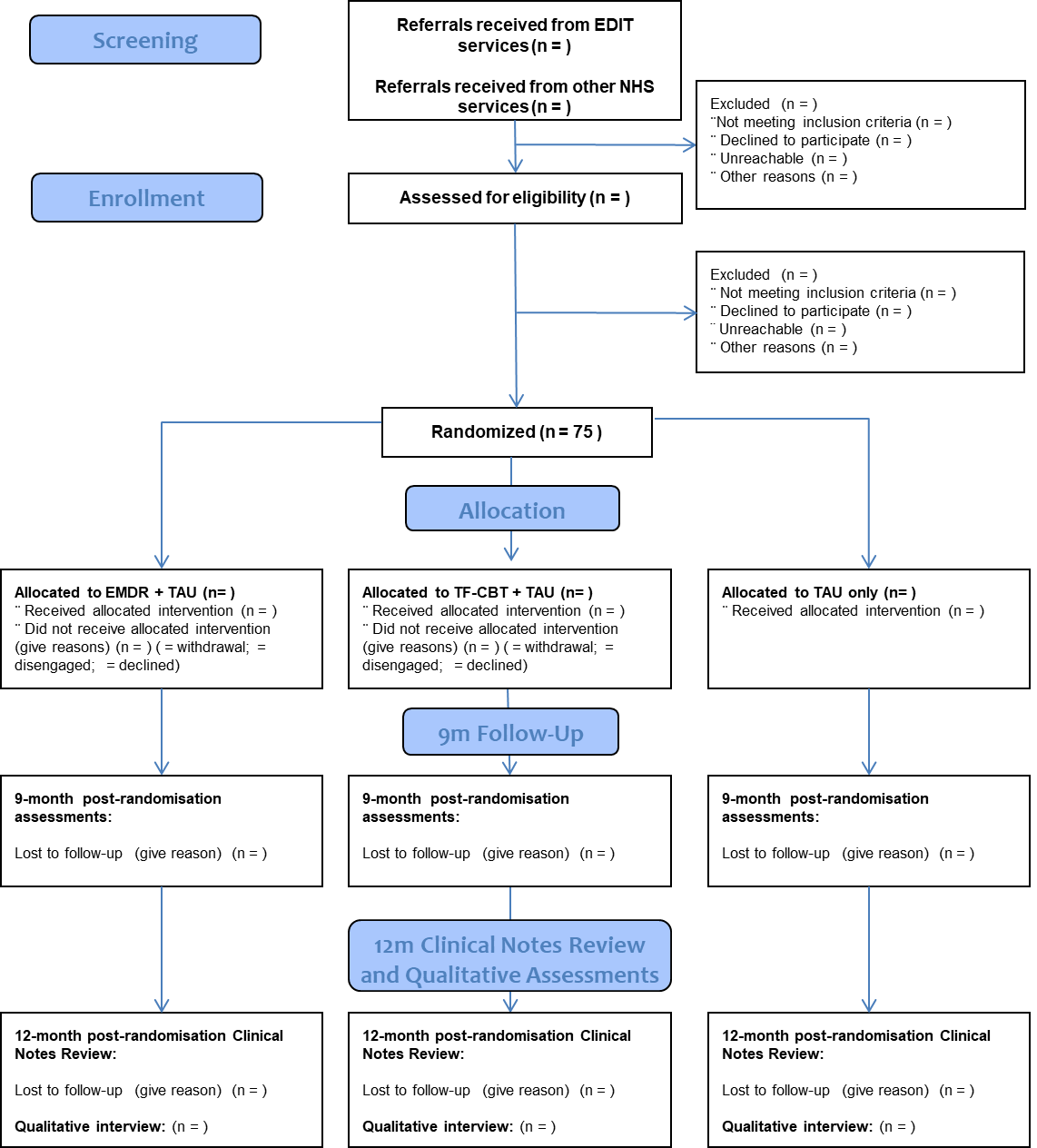

Supplement: Supplementary file 1 — Appendix S1: CONSORT flow diagram. [file EIP-19-0-s001.docx]
